# Supplementary figures and images for: Estimating genetic kin relationships in prehistoric populations
Source: PLoS One. 2018 Apr 23;13(4):e0195491. doi: 10.1371/journal.pone.0195491 (PMC5912749; doi:10.1371/journal.pone.0195491)

**A****All Relationships**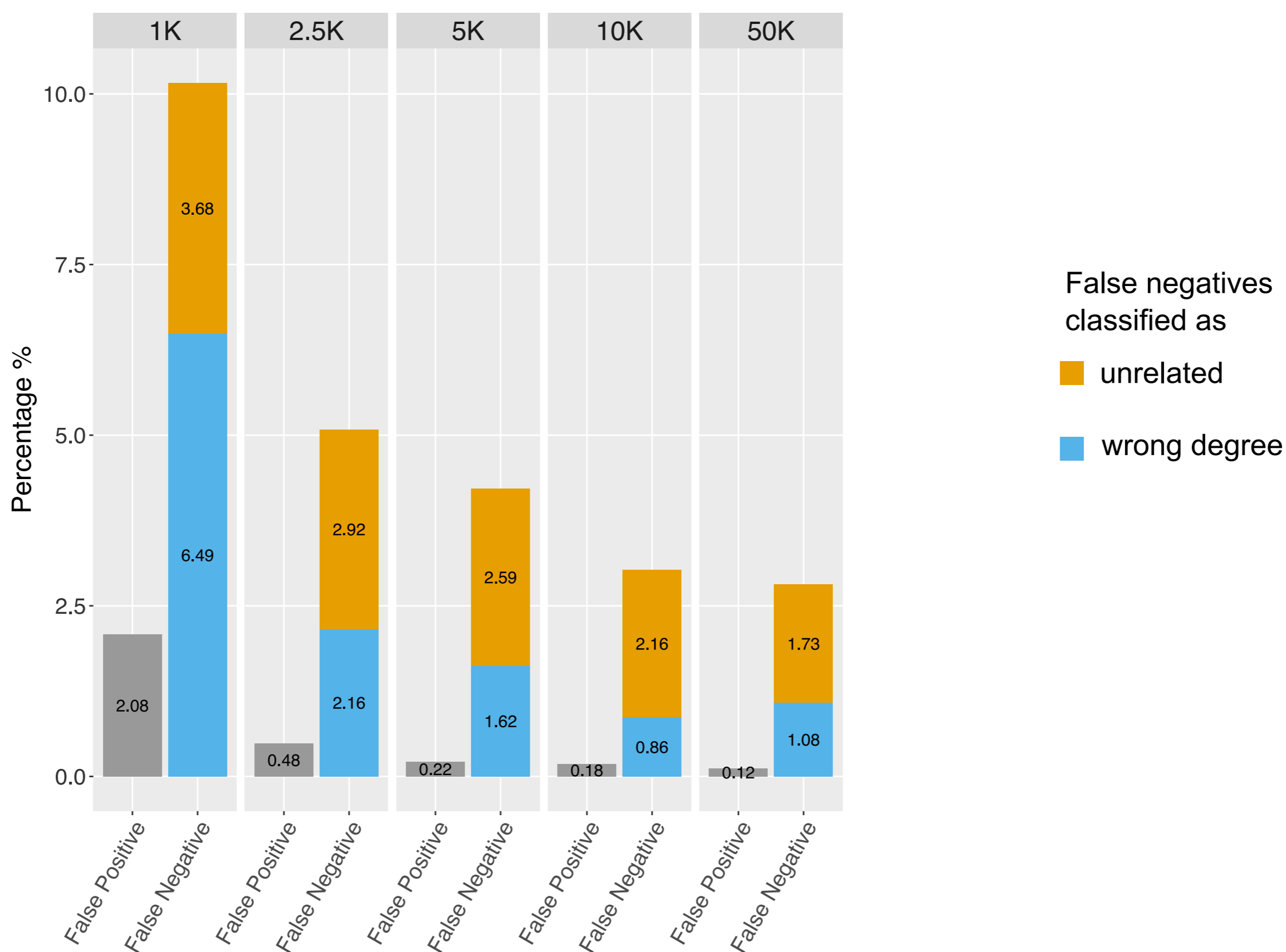**B****First Degree Only**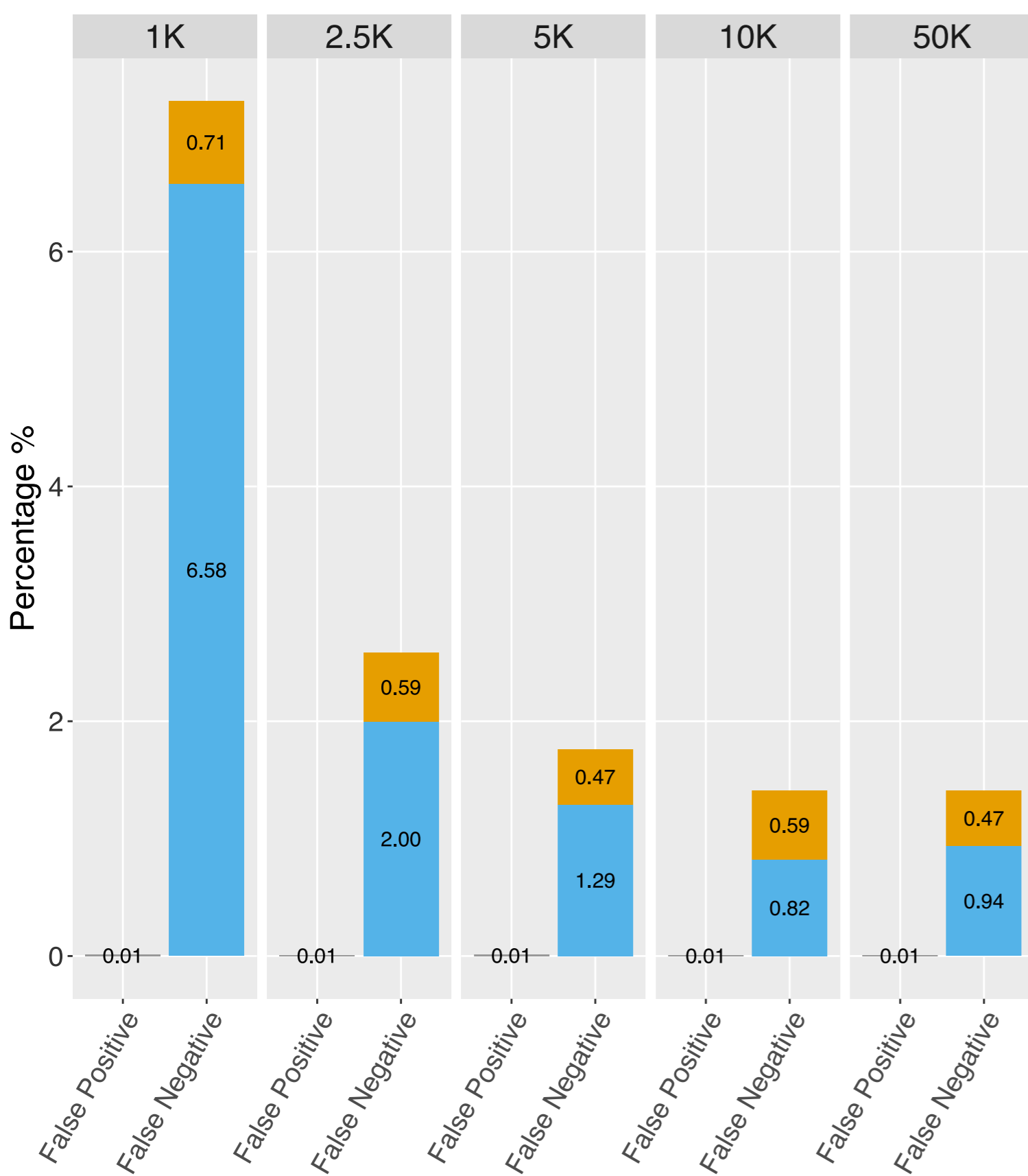**C****Second Degree Only**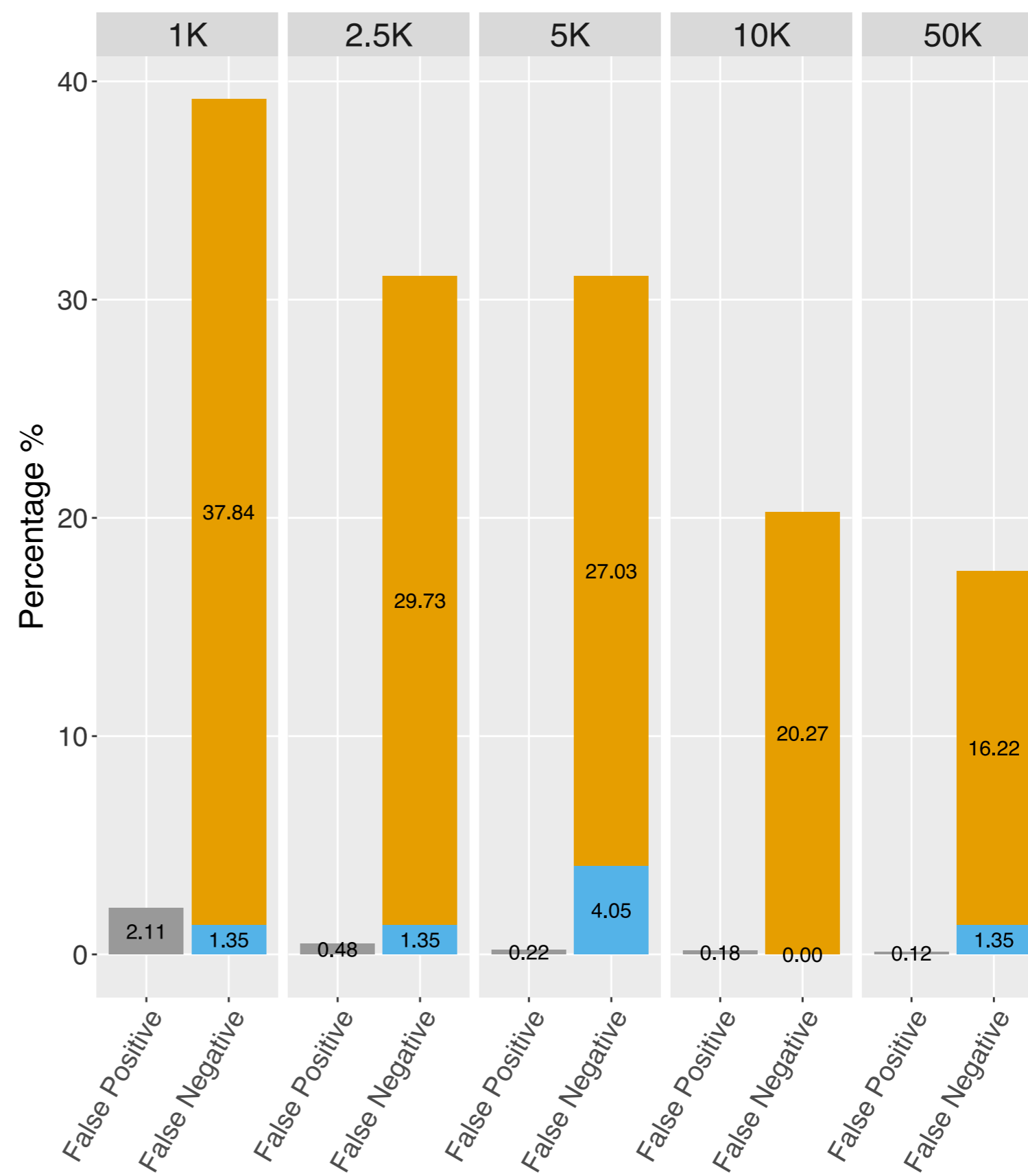

Supplement: S1 Fig — Compare Fig 2. (PDF) [file pone.0195491.s001.pdf]
