# Supplementary material for: Estimating genetic kin relationships in prehistoric populations
Source: PLoS One. 2018 Apr 23;13(4):e0195491. doi: 10.1371/journal.pone.0195491 (PMC5912749; doi:10.1371/journal.pone.0195491)
Supplement: S2 Fig — Compare Fig 3. (PDF) [file pone.0195491.s002.pdf]

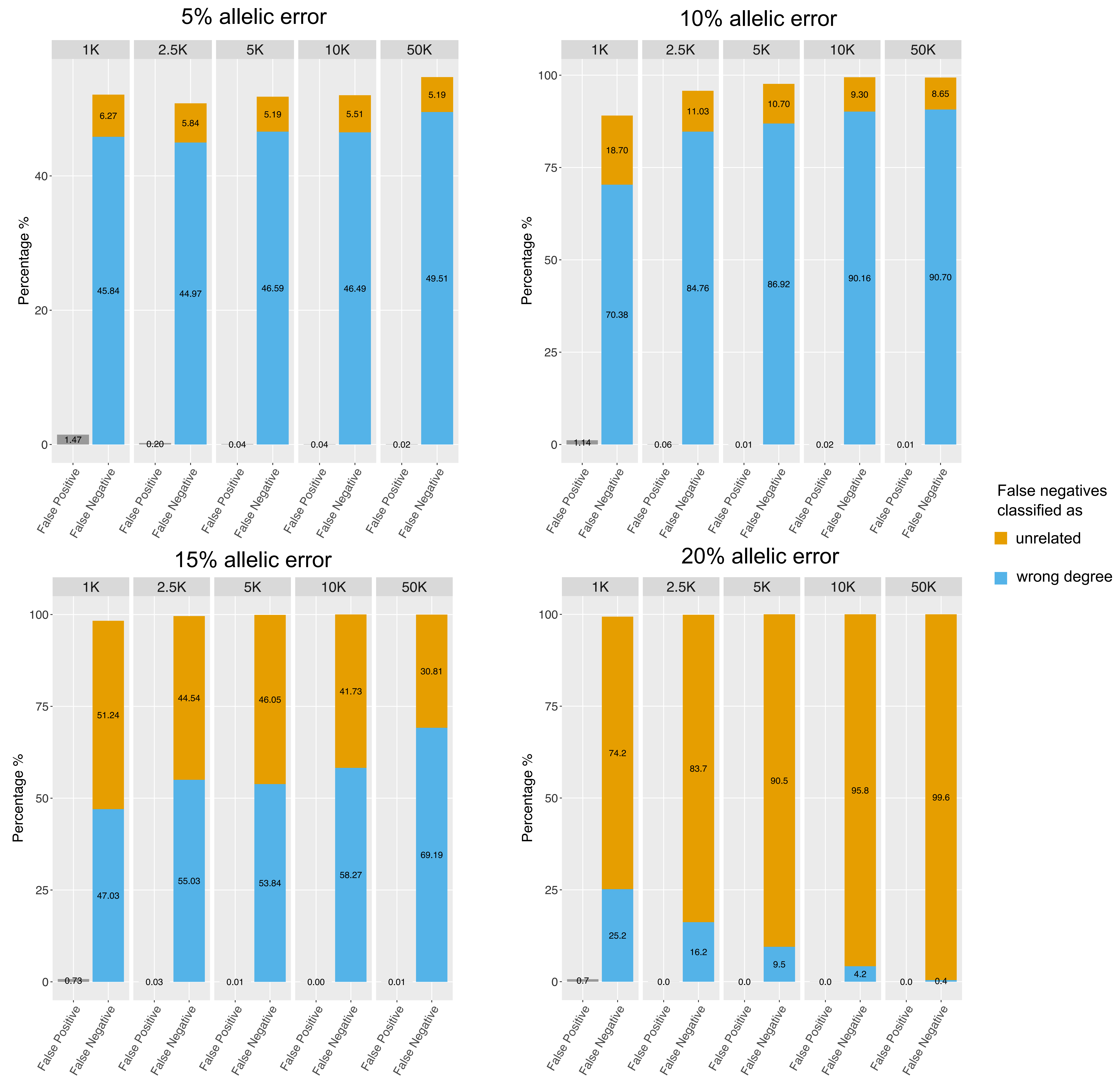
5% allelic error
10% allelic error
15% allelic error
20% allelic error
1K
2.5K
5K
10K
50K
Percentage %
False Positive
False Negative
False negatives classified as
unrelated
wrong degree
1.47
6.27
45.84
0.20
5.84
44.97
0.04
5.19
46.59
0.04
5.51
46.49
0.02
5.19
49.51
1.14
18.70
70.38
0.06
11.03
84.76
0.01
10.70
86.92
0.02
9.30
90.16
0.01
8.65
90.70
0.73
51.24
47.03
0.03
44.54
55.03
0.01
46.05
53.84
0.00
41.73
58.27
0.01
30.81
69.19
0.7
74.2
25.2
0.0
83.7
16.2
0.0
90.5
9.5
0.0
95.8
4.2
0.0
99.6
0.4
