# Supplementary material for: Estimating genetic kin relationships in prehistoric populations
Source: PLoS One. 2018 Apr 23;13(4):e0195491. doi: 10.1371/journal.pone.0195491 (PMC5912749; doi:10.1371/journal.pone.0195491)
Supplement: Supplementary file 3 [file pone.0195491.s006.doc]

2500 SNPs	False Negative Rate (%)	23.3333333333	96.6666666667	0	0
	Incorrect Related (%)	76.6666666667	3.3333333333	64.2857142857	0
	Correct Related Predictions (%)	99.9764595104	100	35.7142857143	100
5000 SNPs	False Positive Rate (%)	0.0235404896	0	0	13.3477633478
	False Negative Rate (%)	6.6666666667	93.3333333333	0	0
	Incorrect Related (%)	90	6.6666666667	82.1428571429	0
	Correct Related Predictions (%)	3.3333333333	0	17.8571428571	100
10000 SNPs	False Positive Rate (%)	0.0235404896	0	0	4.2809042809
	False Negative Rate (%)	6.6666666667	100	0	0
	Incorrect Related (%)	93.3333333333	0	85.7142857143	0
	Correct Related Predictions (%)	0	0	14.2857142857	100
50000 SNPs	False Positive Rate (%)	0.0235404896	0	0	0.0481000481
	False Negative Rate (%)	3.3333333333	100	0	0
	Incorrect Related (%)	96.6666666667	0	100	0
	Correct Related Predictions (%)	0	0	0	100

**5000 SNPs**	False Positive Rate (%)	0	0	0	3.6727272727
	False Negative Rate (%)	4.1666666667	91.6666666667	0	0
	Incorrect Related (%)	95.8333333333	8.3333333333	80	4
	Correct Related Predictions (%)	0	0	20	96
**10000 SNPs**	False Positive Rate (%)	0	0	0	0.6909090909
	False Negative Rate (%)	0	95.8333333333	0	0
	Incorrect Related (%)	100	4.1666666667	84	0
	Correct Related Predictions (%)	0	0	16	100
**50000 SNPs**	False Positive Rate (%)	0	0	0	0
	False Negative Rate (%)	0	100	0	0
	Incorrect Related (%)	100	0	96	0
	Correct Related Predictions (%)	0	0	4	100
